# Supplementary material for: Diamond growth from organic compounds in hydrous fluids deep within the Earth
Source: Nat Commun. 2019 Oct 30;10:4952. doi: 10.1038/s41467-019-12984-y (PMC6821813; doi:10.1038/s41467-019-12984-y)
Supplement: Supplementary file 2 — Supplementary Information [file 41467_2019_12984_MOESM2_ESM.pdf]

SUPPLEMENTARY INFORMATION

for **Organic compounds promote diamond formation in hydrous fluids deep within the Earth**

by Maria Luce Frezzotti

*Corresponding author. Email: maria.frezzotti@unimib.it

Supplementary Note 1 - Details on diamond-bearing fluid inclusions

For the present study, analysed diamond-bearing fluid inclusions are primary - trapped at peak metamorphic conditions (T= 600°C and P > 3.2 GPa; ref. 1; 2; 3) - and distributed inside the garnet cores along with carbonate and coesite/quartz inclusions. Fluid inclusions (< 3 – 20 µm in size) are dominantly aqueous, and two or three phases (L+S ± V) at room temperature. Daughter mineral phases (10-50 vol.%) consist of magnesite, Mg-calcite/calcite, rutile, quartz, and rare diamond, sulphate, and paragonite (Supplementary Table 1). Microthermometry was performed in a few among the largest inclusions (> 10-15 µm in size). On cooling, fluid inclusions freeze at temperatures below -70°C, suggesting the presence of Ca among the anions in the aqueous solution. Although it was not possible to record eutectic temperatures, advanced ice melting at about -20°C indicates the fluid system as a NaCl-KCl-$CaCl_2$ solution. Ice final melting temperatures (Tm) were recorded between -5 and -1°C. Corresponding fluid salinities are calculated between 8 and 3 wt.% in NaCl eq.

In fluid inclusions, the amount of precipitated carbonates greatly exceeds the amount of diamonds and suggests an oxidized nature of trapped fluids. Previous thermodynamic modelling in C-O-H systems[S3] suggested carbon saturation at peak metamorphism in a fluid composition at the water maximum ($0.997 > X_{H_2O}^{fluid} > \sim 0.992$). Mg-calcite/calcite and rutile appear to have precipitated with diamond at P-T peak metamorphic conditions. Quartz could represent SiO_2 saturation at peak conditions (e.g., inversion from coesite), or at later stage cooling and decompression at quartz stability conditions. The high amount of daughter minerals allows calculating hydrous fluid densities > 1.2 g/cm^3 in fluid inclusions without a vapour bubble (ref. 4), consistent with expected extremely high fluid densities at proposed *P-T* trapping conditions. In fluid inclusions, the presence of a vapour bubble is considered resulting from fluid-density decrease due to inclusions stretching by overpressure of the fluid contained in inclusions during the retrograde *P-T* evolution. Raman mapping (Supplementary Fig. 1) reveals strain features in

the host garnet [5,6] around fluid inclusions (e.g., distortion of octahedral Mn^{3+}), and argues that fluid inclusion stretching might have occurred.

Supplementary Note 2 - Details on the characterization of fluid precipitated diamonds from Raman spectra

Raman analysis allows identifying diamonds based on size, structure, sp^3/sp^2-bonded carbon fractions, and surface chemistry. All Raman spectra were collected inside fluid inclusions; in fact, diamonds were analyzed within the aqueous solution from which they precipitated. The spectral parameters of micro- and nano-sized (<1µm) diamonds found in this study are reported in Tables 1, 2, and 3.

Diamond. The Raman features of sp^3-bonded carbon contained in fluid inclusions correspond to crystalline diamond. Diamonds were unambiguously identified by their single peak at 1332 cm^{-1} defined as a three-fold degenerate, zone-centre mode[7,8]. Diamonds inside fluid inclusions show spectral features similar to those of diamonds enclosed in garnet[S1] (Table 1), with the exception that most spectra have relatively weaker intensities, suggesting smaller sizes than the Raman excitation spot of 1 µm. Spectra can show a shifting of the single diamond peak between 1334 and 1328 cm^{-1}, and broad and variable full width at half peak maximum (FWHM) from 4 to 10.6 cm^{-1} (Table 1). The variable shift and broadening of the main peak position are indicative of a decrease in the diamond size associated with stress and strain induced by lattice parameter variations, dislocations, and fractures. Two additional peaks of similar weak intensity at about 1284 and 1300 cm^{-1} forming a triplet with the diamond peak are observed in a few spectra. These two peaks are compatible with assignments reported for metastable (e.g., hexagonal) diamond polytypes[9,10,11].

Poorly ordered sp^2-bonded carbon. In micro-sized diamond spectra (1-2 µm), two bands are often present in the background centered in the regions 1348-1380 and 1576-1600, cm^{-1}, respectively (Fig. 2 and Table 2). Bands are broad, and center position corresponds with the so-called G and D bands of sp^2-bonded carbon[12,13]. The G-band involves the in-plane bond-stretching of pairs of sp^2-bonded carbon atoms originating at all sp^2 sites and does not call for the presence of six-fold rings[S14]. The D (i.e., disorder) band results from the breathing modes of sp^2 sites in rings and its activation involves defects. With increasing disorder in sp^2-structured carbon, the G and D bands broaden, and the relative intensity of the D band increases. In the present spectra, the D and G bands are broader than those in metamorphic graphite[15,16,] even with low crystallinity. D band is generally much wider than the G band (Table 2). The D band FWHM is comprised between 57 and 226 cm^{-1}, whereas the FWHM of the G band ranges from 50 to 98 cm^{-1}. The FWHM of the G band can be used to have indication on the amount of structural disorder in sp^{-2} bonded carbon, since it varies from 10 up to > 100 cm^{-1} from crystalline graphite to amorphous carbons[14].

Thus, G and D bands features correspond to disordered nano-crystalline graphite clusters with a bonding structure similar to that of amorphous sp^2 carbon[14,17]. The general absence of the second-order spectrum of crystalline graphite in the region between 2500 and 3000 cm^{-1} provides further evidence for the presence of a rather disordered sp^2-bonded carbon structure. Sp^2-bonded carbon fraction on diamond surfaces is calculated between 0.01 and 4.9 %, with most values not exceeding 1 % (cf., Methods and Table 3).

Nano-sized diamonds (< 1 µm) generally show a higher fraction of poorly ordered sp^2-bonded carbon at the surface, calculated between 3.6 and 8.6 % (cf., Methods and Extended Data Table 4). A distinctive feature of nano-sized diamond spectra is that the intensity of the diamond peak is weak or even absent (Fig. 2a-c), while the D and G bands have a relatively higher intensity. For sp^2-carbon fractions greater than 3.5 %, the intensity of the D band can obscure in part the considerably less intense diamond peak. Furthermore, the main diamond peak is variably

shifted (1334 - 1328 cm^{-1}) with respect to diamond center position (1331.8 cm^{-1}) and shows a band at around 1220 cm^{-1} appearing as a shoulder (Table 2). The attribution of this band is disputed, although most authors agree that it can originate from domains in diamond separated by defects, or from nanodiamonds[18,19,20]. Besides the G and D modes of disordered sp^2-bonded carbon, a band at a position variable from 1522 to 1566 cm^{-1} is attributed to an amorphous mixture of carbon in sp^2 and sp^3 bonding configuration[21] (Table 3). Band position deviations further suggest variable amounts of H incorporated as multiple C-H bonds in amorphous carbon.

Surface functional groups: Nano-sized diamonds with sp^2-bonded carbon at the surface show terminations by organic functional groups (Table 2). Typical Raman spectra can contain up to 16 bands in the regions 1150-1160, 1440-1480, 1214-1332, 1348-1380, 1520-1565, 1576-1600, 1720-1870, and 2840-2930 cm^{-1}. The bands at around ∼1332, 1214-1226, and 1282-1300 cm^{-1} correspond to diamond features, whereas those at 1348-1380, and 1576-1600 cm^{-1} correspond to D and G bands in sp^2-carbon, respectively (see above). Two bands, in the region from 1720 to 1865 cm^{-1} arise from the stretching vibrations from carbonyl groups. The first band centered at around 1724 cm^{-1} corresponds to the C=O stretching mode in carboxyl radicals, while the latter centered around 1858 cm^{-1} to the C=O stretching mode in carboxylate anions[22].

The bands in the region between 2840 and 3000 cm^{-1} originate from sp^3-hybridized $(CH_x)_n$ (x=2,3) functional groups. Methylene radicals $(CH_2)_n$ are revealed by C-H stretching modes at around 2850 and 2920 cm^{-1}, whereas C-H stretching of methyl radicals $(CH_3)_n$ is recorded around 2880 and 2960 cm^{-1} (Ref. 23,24). A band around 2896 cm^{-1} arises from $(CH_x)_n$ group Fermi resonance vibrations. Bands associated with C-H stretching modes in methane, benzene rings, or other aromatic hydrocarbons are absent. Two bands at 1150 (C-H bending) and 1440 cm^{-1} (C-C stretching) correspond to transpolyacetylene segments $(CH)_n$ (Ref. 14,24,25) on diamond surfaces

Supplementary Figure 1 – Raman map of garnet surrounding fluid inclusions.

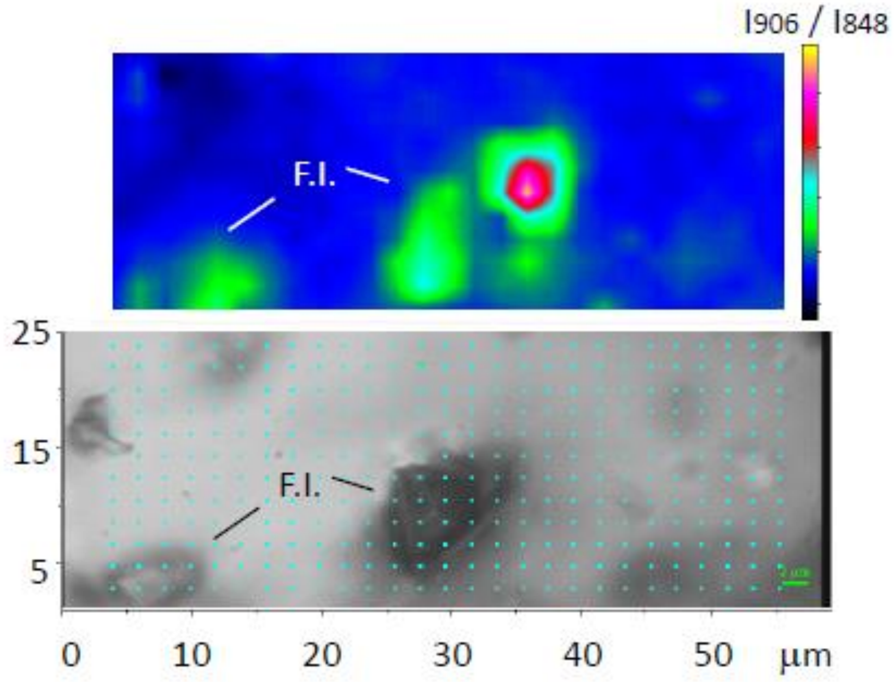

Top - Raman map illustrating the variations of intensity ratios (I_{906}/I_{848}) between the (Si-O) symmetric stretching mode (A1g; 906 cm^{-1}) and (Si-O) asymmetric stretching mode (T1g; 848 cm^{-1}) as I_{906} / I_{848} ratio in garnet close to a fluid inclusion. It shows the progressive hardening of the symmetric stretching mode at 906 cm^{-1} of SiO_4 groups in garnet (colour scale from black to yellow) in areas adjacent to the inclusion. Hardening of A1g mode intensity in spectra results from a reduction of the symmetry patterns due to high strain in these regions. Bottom – B&W microphotograph of the garnet region containing fluid inclusions (F.I.) mapped by Raman spectroscopy.

Supplementary Table 1 | Fluid inclusion characteristics as derived from selected Raman anayses

Sample	Garnet	Fluid inclusion		Daughter minerals				
n.	mode harden.	n.	size µm	Carbon		Carbonate	Silicate	Oxide
G2C	A	D2	10	diamond		calcite		rutile
G2C	A	D5	18	diamond	Sp^2-C	Mg-calcite	quartz, parag.	
G2C	A	D6	15	diamond		calcite		
G2C	A	E1	5		Sp^2-C			
G2C	A	E4	6	diamond	Sp^2-C	carbonate ions	quartz	
G2C	A	E5	6	diamond	Sp^2-C	carbonate		
G2C	A	E7	4	diamond		calcite		
G2C		E8	4	diamond		carbonate		
G2C		G9	5	diamond		carbonate		
G2G		F2	12	diamond		calcite	quartz	rutile
G2G		M5	8	diamond		calcite		rutile
G2G	A	M6	10	diamond	Sp^2-C	carbonate ions		rutile
G2B	A	BA8	16	diamond		Mg-calcite		rutile
M2B	A	B2	5	diamond				
M2B	A	B3	4		Sp^2-C			
M2B	A	B4	4		Sp^2-C			
M2B	A	B6	7	diamond	Sp^2-C	calcite		
M2B		C1	20	diamond	Sp^2-C	calcite		rutile
M2B	A	C5	4	diamond	Sp^2-C	carbonate?		
M2B	A	C7	10	diamond	Sp^2-C			rutile
D2A	A	B1	6	diamond	Sp^2-C			
ALC2	A	1	6		Sp^2-C	Mg-calcite		
ALC2	A	B5	7	diamond	Sp^2-C	Mg-calcite		rutile
ALC2	A	B6	5	diamond	Sp^2-C		quartz	
M2B	A	C3	12	diamond	Sp^2-C	calcite		rutile
G2C	A	I10	12	diamond	Sp^2-C			
3D2AG		A1	6	diamond	Sp^2-C	calcite		rutile

Mode harden. = Garnet vibration mode hardening

Supplementary References

1 Groppo, C., Beltrando, M. & Compagnoni, R. The P-T path of the ultra-high pressure Lago di Cignana and adjointing high-pressure meta-ophiolitic units: insights into the evolution of the subduction Tethyan slab. *J. Metamorph. Geol.* **27**, 207–231 (2009).

2 Frezzotti, M.-L., Selverstone, J., Sharp, Z. D. & Compagnoni, R. Carbonate dissolution during subduction revealed by diamond-bearing rocks from the Alps. *Nat. Geosci.* **4**, 703-706 (2011).

3 Frezzotti, M.-L., Huizenga, J-M., Compagnoni, R. & Selverstone, J. Diamond formation by carbon saturation in C-O-H fluids during cold subduction of oceanic lithosphere. *Geochim. Cosmochim. Acta* **143**, 68–86 (2014).

4 Bakker, R. J. Package FLUIDS 1. Computer programs for analysis of fluid inclusion data and for modelling bulk fluid properties: *Chem. Geol.* **194**, 3-23 (2003).

5 Hatch D. M. & Griffin D. T. Phase transitions in grandite garnets. *Am. Mineral.* **74**, 151-159, (1989).

6 Hofmeister, A. M., Giesting, P. A., Wopenka, B., Gwanmesia, G. D. & Jolliff, B. L. (2004). Vibrational spectroscopy of pyrope-majorite garnets: Structural implications. *Am. Mineral.* **89**, 132-146.

7 Solin, S. A. & Ramdas, A. K. Raman Spectrum of Diamond. *Phys. Rev. B* **1**, 1687 (1970).

8 Knight, D. S. and White, W. B. Characterization of diamond films by Raman spectroscopy. *J. Mater. Res.* **4**, 385-1989 (2011).

9 Phelps, A. W. “Interstellar Diamond: I. Condensation and Nucleation”, Lunar and Planetary Science XXX Meeting, Abstract 749 (1999).

10 Bhargava, S., Bist, H.D., Sahli, S., Aslam, M., & Tripathi, H.B. Diamond Polytypes in the chemical vapor deposited diamond films, *Appl. Physic Lett.* **67**, 1706-1708 (1995).

11 Wang, Z., Zhao, Y., Zha, Ch-S., Xue, Q. & Downs, R. T. X-Ray. Induced Synthesis of 8H Diamond. *Adv. Mater.* **20**, 3303-3307 (2008).

12 Tuinstra, F. & Koenig, J. L. Raman spectrum of graphite. *J. Chem. Phys.* **53**, 1126-1130 (1970).

13 Robertson, J. Diamond-like amorphous carbon, *Mater. Sci. Eng. Rep.* **37**, 129-281 (2002)

14 Ferrari, A. C., & Robertson, J. Resonant Raman spectroscopy of disordered, amorphous, and diamondlike carbon. *Phys. Rev. B* **63**, 121405 (2001).

15 Beyssac, O., Goffé, B., Chopin, C. & Rouzaud, J. N. Raman spectra of carbonaceous material in metasediments; a new geothermometer. *J. Metam. Geol.* **20**, 858-712 (2002).

16 Perraki, M., Proyer, A., Mposkos, E., Kaindl, R., & Hoinkes, G. Raman microspectroscopy on diamond, graphite and other carbon polymorphs from the ultrahigh-pressure metamorphic Kimi Complex of the Rhodope Metamorphic Province, NE Greece. *Earth Planet. Sci. Lett.* **241**, 672-685 (2006).

17 Foustoukos, D. Metastable equilibrium in the C-H-O system: Graphite deposition in crustal fluids. *Am. Mineral.* **97**, 1373-1380 (2012).

18 Obraztsova, E. D. et al. Raman and photoluminescence investigations of nanograined diamond films. *Nanostruct. Mater.* **6**, 827–830 (1995).

19 Yushin, G. N., Osswald, S., Padalko, V. I., Bogatyreva, G. P. & Gogotsi, Y. Effect of sintering on structure of nanodiamond. *Diamond Relat. Mater.* **14**, 1721-1729 (2005).

20 Osswald, S., Mochalin, V. N., Havel, M., Yushin, G. & Gogotsi, Y. Phonon confinement effects in the Raman spectrum of nanodiamond. *Phys. Rev. B* **80**, 075419 (2009).

21 Ferrari, A. C. & Robertson, J. Interpretation of Raman spectra of disordered and amorphous carbon. *Phys. Rev. B* **61**, 14095–14107 (2000).

22 Génin, F., Quilès, F. & Burneau A. Infrared and Raman spectroscopic study of carboxylic acids in heavy water. *Phys. Chem. Chem. Phys.* **3**, 932-942 (2001).

23 Mcnamara, K. M., Levy, D. H., Gleason, K. K. & Robinson, C. J. Nuclear magnetic resonance and infrared absorption studies of hydrogen incorporation in polycrystalline diamond. *Appl. Phys. Lett.* **60**, 580 (1992).

24 Dischler, B., Wild, C., Mullersebert, W. & Koidl, P. Hydrogen in polycrystalline diamond - an infrared-analysis. *Physica B* **185**, 217 (1993).

25 Kuzmany, H. Pfeiffer, R. Salk, N. & Gunther, B. The mystery of the 1140 cm^{-1} Raman line in nanocrystalline diamond films. *Carbon* **42**, 911 (2004).
